# Use of porous high-density polyethylene grafts in open rhinoplasty: no infectious complication seen in spreader and dorsal grafts

**DOI:** 10.1186/1746-160X-10-52

**Published:** 2014-12-22

**Authors:** Shabahang Mohammadi, Mohammad Mohseni, Masoumeh Eslami, Hessein Arabzadeh, Morteza Eslami

**Affiliations:** Ear Nose Throat (ENT) and Head and Neck Surgery Research Center, Hazrat RasoulAkram Hospital, Iran University of Medical Sciences, Sattarkhan St, Tehran, Iran; ENT Department of Firouzgar Hospital, Medical student of Iran University of Medical Sciences, Vali Asr Street, Tehran, Iran

**Keywords:** Medpor complication, Rhinoplasty, Spreader graft, Dorsal graft

## Abstract

**Objective:**

The aim of this study is to use porous high-density polyethylene grafts (Medpor) in open rhinoplasty and then assess complication rate and aesthetic outcomes.

**Methods:**

In a prospective cohort study, we performed open rhinoplasty and employed Medpor as rhinoplasty grafts. Then we compared their complication rate.

**Results:**

In a total of 64 patients, 84 Medpor grafts -38 dorsal grafts, 23 strut grafts, 8 rim grafts, 5 button grafts and 10 spreader grafts – were utilized. Moreover, 5septal perforation repairs with Medpor were performed. The complication rates were 5.3% in dorsal graft (complication in dorsal graft was only movement of implant), 21.7% in strut graft and 25.0% in rim graft. No complication was seen in spreader and button grafts. All 5septal perforation repairs were successfully performed with the same rhinoplasty approach.

**Conclusion:**

Medpor can be used as dorsal and spreader graft in reconstruction of severe nose deformity with lowest complication rate and without infectious complication and extrusion.

## Introduction

In 1970s porous high-density polyethylene (PHDPE, Medpor) was introduced by its very important advantage that was minimal foreign body reaction [1]. Medpor are made of biocompatible porous polyethylene material with the interconnecting pore structure allows for fibrovascular in-growth and integration of patient's tissue [2]. Upon introduction, use of these implants in rhinoplasty for reconstruction of a nose with severe deformity was a great success, especially in revision rhinoplasty of saddle and deviated nose [3–5]. However, nowadays this practice is limited, duo to potential complications [6, 7].

Plastic surgeons and ENT specialists are always in need of a strong and safe support when dealing with sever nose deformities like deviated and saddle noses. Bone and cartilage grafts both are lacking, as bone grafts may resorb and cartilage grafts may return the deformity because of cartilage memory [3, 8].

It seems study of Medpor grafts and finding the ones with minimum complications after treating severe nose deformities will be helpful. The aim of this study is to use Medpor in open rhinoplasty and then assess complication rate and aesthetic outcomes.

## Methods and materials

### Ethical approval

This study was a prospective cohort study approved by the institutional review board of the ENT research center of Rasool-e-Akram hospital, Iran University of Medical Sciences (IUMS) and started on 2008. We thoroughly explained all the available grafts, and advantages and disadvantages of each one of them to the patients. Only the patients who gave their content to Medpor were included in the study. Although the patients were aware of possible complications such as extrusion, movement, fistule and infection, most of them selected Medpor because of its rigidity and not having to experience the complications of donor site. Patients were not charged for Medpor grafts or any revision surgery. any surgical procedures was also included in the consent form signed by patients or their guardians. Also another written informed consent was obtained from the patients for publication of the clinical data and any accompanying images. A copy of the written consent is available for review by the Editor of this journal.

### Patients

Patients with deviated or saddle nose, who underwent a previous rhinoplasty and patients with severe pinching in supra-alar region, were admitted in this study. In all of the patients, septal cartilage was insufficient due to prior surgery (septoplasty or rhinoplasty), nasal trauma or septal cartilage damage. Patients with underlying diseases especially vasculitis and other condition that may disrupt blood supply such as smoking, were excluded of the study.

The clinical characteristics included gender, age, types of grafts were used and complications which were entered to a self-designed check list before and after surgery.

### Surgical techniques

In our study, we used open rhinoplasty procedure and employed Medpor in all 64 patients. In these patients, we used Medpor as columellar strut, dorsal, spreader, rim and batten grafts.

**Figure 1:**
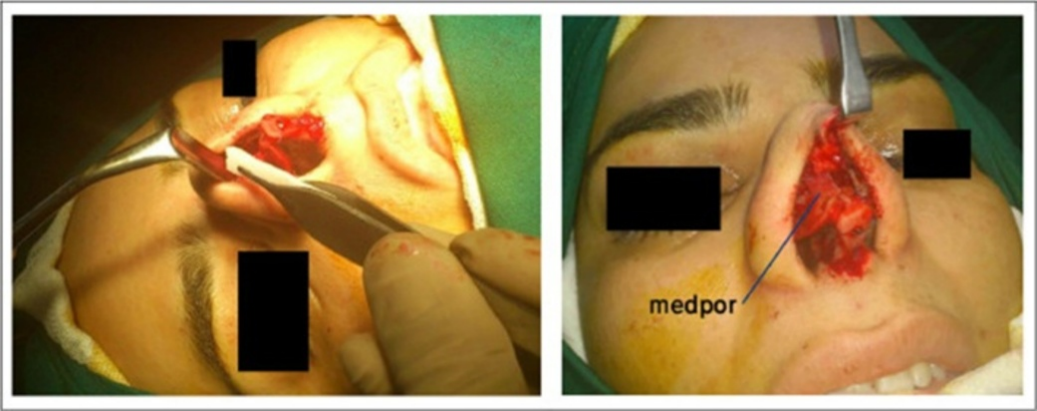
**Dorsal Medpor graft insertion.** In a patient with saddle nose deformity.

**Figure 2:**
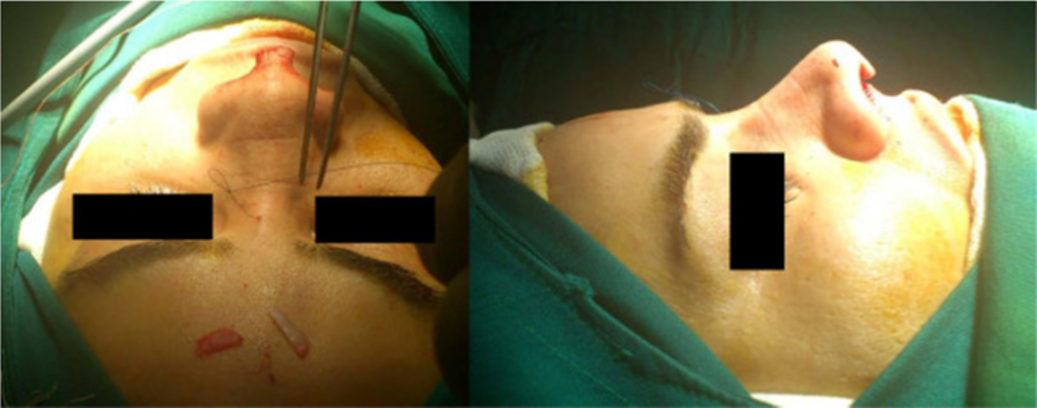
**Fixatfion of graft.** Medpor were fixed to the skin with 5–0 nylon.

In case of strut grafts, the plates were fixed with 6–0 nylon to the medial crura of both lower lateral cartilages, to have good projection and rotation in the patients with tip ptosis.For dorsal grafts, the plates were inserted in dorsum in subperiosteal plane (Figure 1), in suitable size and height (in some patients with severe saddle nose, Medpor was inserted in 2 or 3 layers), and then were fixed to the skin with 5–0 nylon (Figure 2). The sutures were removed one week later. In patients with thin skin we used temporalis fascia and covered Medpor with it.

In case of spreader grafts, the plates were inserted and fixed between upper lateral cartilage and septum with nylon 5–0 to correct the deviation or ULC (upper lateral cartilage) pinching.

We also used Medpor as rim grafts. We first inserted the plates in pocket caudal to the LLC (Lower Lateral Cartilage) through marginal incision and then sutured the incision. In addition, we put Medpor as batten graft overlay LLC through marginal incision. These techniques were used to augment the cartilage in patients with pinching of LLC or severe cartilage weakness.

In patients with septal perforation, due to trauma or prior septoplasty, we repaired the perforation in the same rhinoplasty procedure. Bilateral mucoperichondrium was elevated completely. Then, the nasal floor mucoperiosteum was elevated up to the inferior turbinate. At last, Medpor of adequate size was inserted between septum and left-sided mucoperichondrium. We tried to close the mucosal defect primarily with 4–0 vicryl on Medpor.In the surgery procedure, we avoided direct handling of Medpor (Figure 1); and implant irrigated with antibiotic solution (gentamycin) before and after implant insertion. Also in all patients, insertion of implant performed without any tension of overlying skin to minimize the risk of extrusion.

In all patients perioperative infusion of Cefazolin IV performed; followed by oral cefalexin for 1 week.

### Follow-up

In this study, the surgeons followed up the patients for 3 years in monthly intervals and checked for extrusion, Infectious symptoms (erythema, fistula, and abscess), implant movement, and aesthetic outcomes. Typically, in patients without complication, digital standard photography was performed 1 year after surgery.

## Results

**Figure 3:**
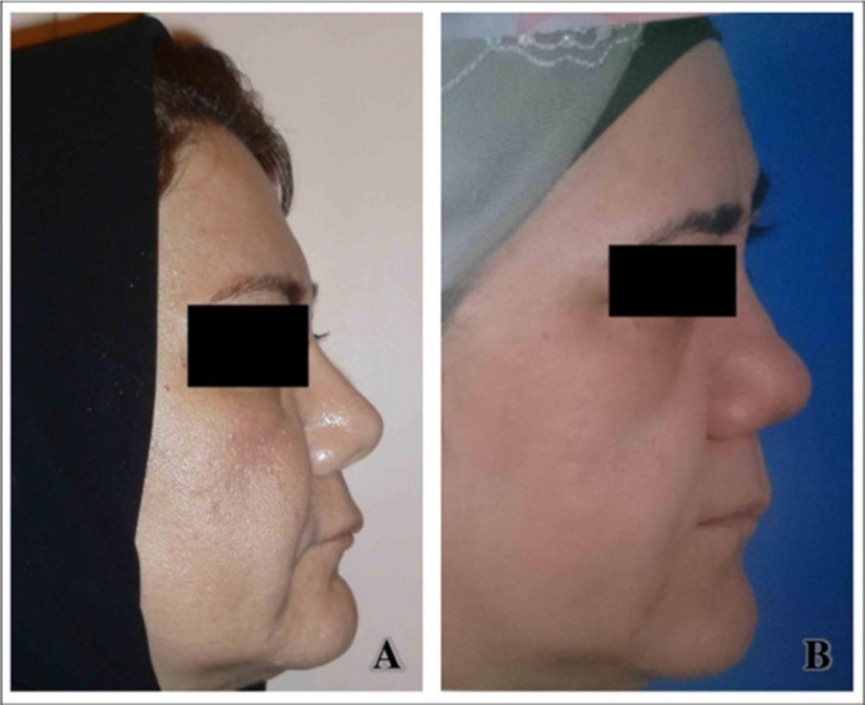
**Medpor insertion as dorsal graft for reconstruction of mild saddle nose (supra tip depression).** Successful results in a 32-year-old woman one year after surgery (**A** = before surgery, **B** = after surgery).

**Figure 4:**
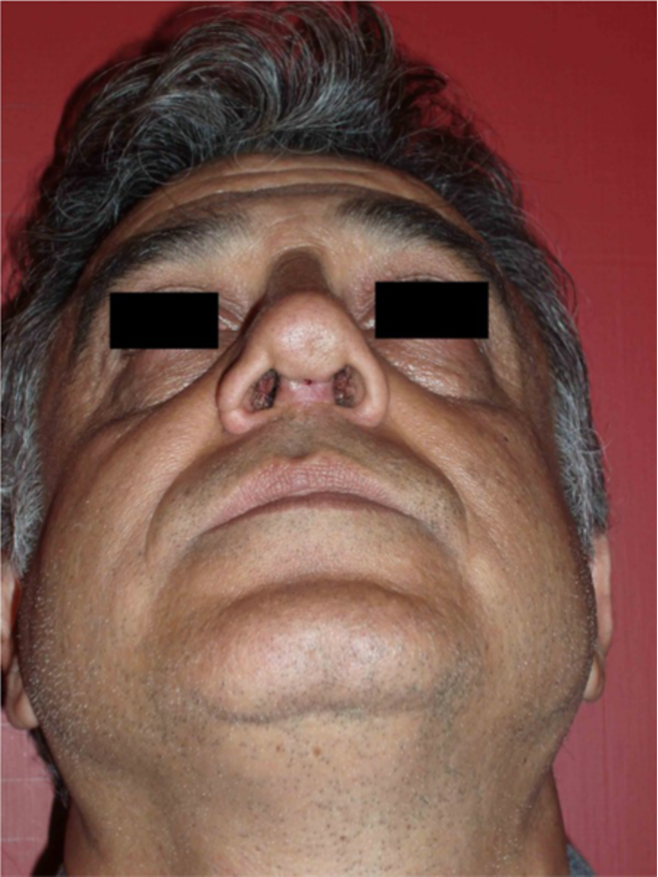
**Columella fistula in a 42 years old man with strut Medpor graft.** 45 days after surgery.

**Figure 5:**
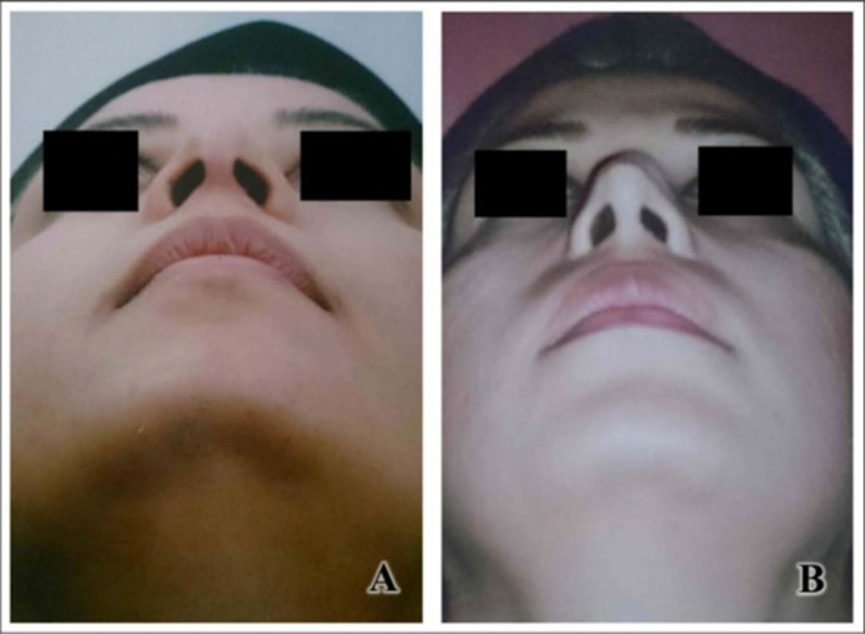
**Deviation of strut Medpor graft in a 28-year-old woman.** 3 month after surgery (**A** = before surgery, **B** = after surgery).

In a total of 64 patients, 84 Medpor grafts – 38 dorsal grafts, 23 strut grafts, 8 rim grafts, 5 button grafts and 10 spreader grafts – were utilized. Moreover, 5 septal perforations repairs with Medpor were performed (Table 1). Age of the patients ranged from 15 to 58 years with an average of 36 ± 6 years. 39 patients were women and 25 were men.Among 38 patients with dorsal graft placement, complications (lateral movement of implant) were seen in 2 patients (complication rate = 5.3%), but no infection symptom or extrusion of implant was seen (Figure 3). Revision surgery and graft removal was needed in one of them. However, the other one had deviated nose before surgery, and this movement of implant was acceptable and surgery was not required.From 23 patients with strut graft insertion, complication was seen in 5 patients, including 3 deviation of implant and tip, and 2 infectious symptoms; 1 fistula formation in culomella and skin thickness and erythema in another without response to antibiotic (complication rate = 21/7%). Revision surgery and implant removal was performed in all 5 patients and replacement with calvarial bone graft or cartilage allograft was performed (Figures 4 and 5).

**Table 1:** **A summary of all the implanted grafts**

The site of graft	N = grafts	N = complication	N = revision surgery
Dorsal	38	2	1
Strut	23	5	5
Rim	8	2	2
Button	5	0	0
Spreader	10	0	0
Total	84	9	8

**Figure 6:**
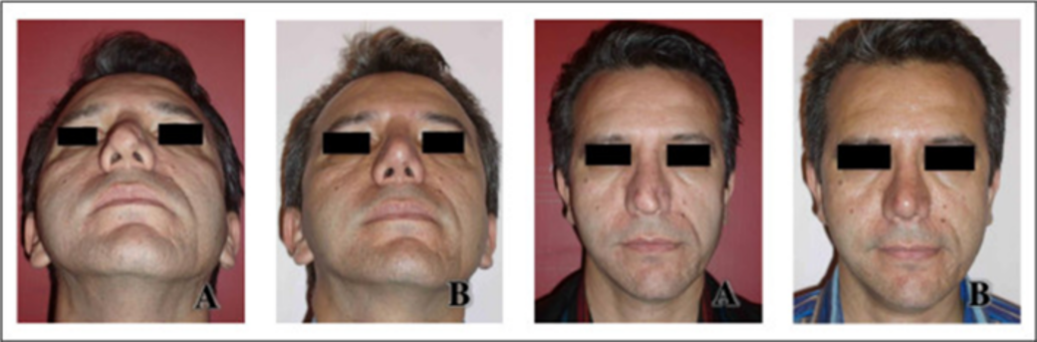
**Medpor insertionas left spreader and button graft for deviation and pinching repair.** Excellent results in a 45-year-old man one year after surgery (**A** = before surgery, **B** = after surgery).

From 8 patients with rim graft insertion, extrusion was seen in 2patients (complication rate = 25%). In contrast, when Medpor was used as button graft, no complication was seen. Spreader graft insertion was performed in 10 patients and no complication was seen in this group either (Figure 6, Table 2).

**Table 2:** **Comparison of complication rates of different rhinoplasty grafts**

Grafts	Movement of implant (N)	Infection (N)	Extrusion (N)	Total rate of complication (%)	Rate of rev surgery (due to complication) (%)
Dorsal	2	-	-	5.3	2.6
Strut	3	2	-	21.7	21.7
Rim	-	-	2	20.0	20.0
Spreader	-	-	-	0	0
Button	-	-	-	0	0
**Total**	**5**	**2**	**2**	10.7*	9.5**

*this indicates the overall rate of complication (9 cases in 84 grafts).

**this is the overall percentage of revision surgery (8 cases in 84 grafts).

In 5 patients who all had previous septoplasty or nasal trauma and saddle nose, septal perforation repair was done in the same rhinoplasty procedure. Size of the perforations was smaller than 2 centimeters. Follow-up physical exams with nasal endoscopy indicate successful repair of perforations without extrusion and other complication.

The time between surgery and onset of infectious complications and extrusion in patients was 25 to 68 days whit mean 37.2 ± 12 days.

## Discussion

Alloplasts and specifically Medpor are used in craniofacial reconstructions for many years [2, 9]. Porous high-density polyethylene grafts (Medpor) do not lead to the problems associated with solid and nonporous grafts. Their physical and chemical properties are more compatible and they are less likely to result in complications. Tiny holes with average size of 150 microns allow maximum in-growth in these grafts. This will prevent movement of the graft and formation of dead spaces, which consequently facilitates migration of inflammatory cells and so reduces infection risks [10].

As these implants are artificial, there are no donor site complications associated with them. In contrast, autogenous rib grafts and bone grafts may have complications due to donor site such as pneumothorax in rib graft harvest and increase of operation time. Due to bone grafts’ natural proportionate, they may resorb and are difficult to shape. Auricle grafts are not considered an appropriate support as they are weak, thin and non-uniform [8, 10].

Medpor can be used as grafts of different size with high biocompatibility to be specifically utilized in reconstruction of deviated noses, saddle deformities and revision surgery rhinoplasty duo to lack of cartilage [3, 5]. However, nowadays employment of alloplastic implants especially Medpor has decreased duo to reports of complications such as extrusion and fistula [6, 7]. Although most surgeons prefer autologous grafts, selection of graft must be individualized for each patients due to history of prior surgery, associated structural abnormalities and characteristics of the overlying skin and soft tissue [11].

Many surgical techniques for correction of deviated noses are reported, but recurrence – duo to cartilage memory and scar contracture – is prevalent. Therefore, a stable, strong and permanent support is required to prevent recurrence as well as granting any desirable shape in one or both side of septum. Medpor implants include excellent contouring, increased mechanical stability, decreased risk of implant migration and infection [12]. They improve nose function and can resist to trauma and scar contracture force. They are also superior to cartilage grafts as they are free of cartilage memory problem and do not lead to recurrence [3].

Emsen et al. [3] utilized Medpor as spreader grafts in 18 patients with deviated nose; none of the patients experienced recurrence or extrusion. Two similar studies proved successful performance of Medpor as the spreader graft [13, 14]. In Our study, Medpor was used as spreader graft in 10 patients with deviated nose and no complications and recurrences were seen in a two-year follow-up. This agrees with the result of previous studies.

Reconstruction of saddle nose is one the challenges in front of ENT specialists. When nasal septal cartilage is available, it is preferred source [15], but in reconstruction of severe cases of saddle nose, septum and auricle cartilages cannot provide the required augmentation. However, Medpor is useful in these situations and is especially effective in revision rhinoplasty where patient cartilage is not adequate [10, 16].

The next option was calvarial bone grafts. Occasionally, patients with calvarial bone grafts do not recognize it as foreign body and they have a better feeling towards it [17]. Nevertheless, most patients reject it because of donor site need [18, 19]. Razmpaet al. used a combination of Medpor and irradiated homograft rib cartilage as dorsal grafts in reconstruction of saddle nose. 2 out of 32 patients experienced implant displacement, but no extrusion was reported [16]. Similarly, our study showed successful usage of Medpor as dorsal graft. There was only a single case of implant movement that required revision surgery, but no extrusion was seen.

In our study, most complications occurred in rim grafts, so we do not recommend the use of Medpor as rim grafts. No complication was reported after use of Medpor as button graft, but the data is not sufficient and we cannot recommend its use. After use of Medpor implants as strut graft, 5 patients experienced complications. Therefore, although Medpor provides a strong support for nose tip, it is not recommended to use it as strut graft either.

Complications associated with Medpor could be categorized into implant displacement, infectious (fistula, erythema and abscess formation) complications and extrusion. Our study indicates, 44.5 percent of the cases (4 out of 9) to be infectious complications and extrusion. Use of Medpor implants as dorsal graft resulted in no infectious complication and extrusion. Most infectious complications were recorded strut grafts and most extrusion was seen in rim grafts. It seems insertion of Medpor close to incision site is a risk factor for extrusion.

In addition to implant site, several factors are reported to increase reaction to Medpor and complication rate. For example, anything that disrupts tissue perfusion such as long term cocaine inhalation and underlying vasculitis such as wegner’s granulomatosis could lead to implant extrusion [10]. In our study any of patients didn’t have these conditions.

Findings of this study are consistent with the results of previous studies. Both this study and the previous studies imply that use of Medpor implants as dorsal and spreader grafts is safe, is less prone to complications and has a high success rate. If the implant is used for appropriate patients who have sufficient tissue perfusion, no underlying disease and thick skin for larger tissue support, extrusion and infectious complication risk factor lessens to near zero and Surgeons could use them with more confidence [3, 5, 13, 14, 16–19]. Although in this study infectious complication and extrusion in dorsal graft was not seen, due to some report of Medpor extrusion in this place in long term follow up [7, 20], we can't disregard this late complication. Use of Medpor for dorsal graft can be based on surgeons and patients preference with knowing of this complication that is less frequent in dorsal graft compared with strut or rim graft.

Nasal septal perforation presents a challenging problem to ENT specialists. Different surgical techniques for the repair of septal perforation have been proposed. Kridel et al. [21] popularized an open rhinoplasty approach for septal perforation repair. This approach provides the surgeon with great exposure, andis advantageousin the repair of septal perforation especially in large and posteriorly located perforations and in patients with revision nasal surgery and lack of cartilage. Septal perforation repair can be safely combined with open rhinoplasty. Some of the routine rhinoplasty maneuvers such as medial osteotomies and hump removal could even facilitate septal perforation repair [22, 23].

The use of titanium mesh and local pedicled mucoperiosteal flap in repairing septal perforation was first described in 2006 [24]. Because most patients with septal perforation have had previous septal surgeries with removal of cartilage, titanium membrane and Medpor offer another advantage in these situations. These alloplasts also help eliminate the risk of saddle nose deformity that is a long-term complication especially in large high septal perforations [22].

In this study, we used Medpor for septal perforation repair successfully without any complication; all of our patients had small size of perforation (lower than 2 cm).

## Conclusion

Minimum complications were reported in use of Medpor implants as dorsal and spreader grafts. Especially in spreader grafts the infectious complication rate was lower. However, most of infectious complications and extrusion were seen after implanting Medpor as strut and rim grafts in our stydy Some complication and extrusion were seen in long term follow up in Medpor dorsal graft in other study and because treatment of infectious complications and skin diseases is complicated, we recommend use of Medpor implants only as spreader graft safely.

Also septal perforation repair with Medpor, can help dorsal augmentation in sever saddle nose deformity and can combine with other graft insertion in open rhinoplasty approach.

## Abbreviations

LLC: Lower lateral cartilage
ULC: Upper lateral cartilage
IUMS: Iran University of Medical Sciences.
