# Erratum to: Use of porous high-density polyethylene grafts in open rhinoplasty: no infectious complication seen in spreader and dorsal grafts

**DOI:** 10.1186/s13005-015-0083-8

**Published:** 2015-10-02

**Authors:** Shabahang Mohammadi, Mohammad Mohseni, Masoumeh Eslami

**Affiliations:** Ear Nose Throat (ENT) and Head and Neck Surgery Research Center, Hazrat RasoulAkram Hospital, Iran University of Medical Sciences, Sattarkhan St, Tehran, Iran

The original version of this article [1] unfortunately contained a mistake. The author list was incorrect. The correct author list is: Shabahang Mohammadi, Mohammad Mohseni and Masoumeh Eslami.
